# The P4 Study: Postpartum Maternal and Infant Faecal Microbiome 6 Months After Hypertensive Versus Normotensive Pregnancy

**DOI:** 10.3389/fcimb.2022.646165

**Published:** 2022-01-31

**Authors:** Daniella Frances Susic, Leanne Wang, Lynne Margaret Roberts, Michelle Bai, Andrew Gia, Emily McGovern, Xiao-Tao Jiang, Gregory K. Davis, Emad El-Omar, Amanda Henry

**Affiliations:** ^1^School of Women’s and Children’s Health, Faculty of Medicine, University of New South Wales, Sydney, NSW, Australia; ^2^Microbiome Research Centre, University of New South Wales, Sydney, NSW, Australia; ^3^Department of Womens and Childrens Health, St. George Hospital, Sydney, NSW, Australia; ^4^Faculty of Medicine, University of New South Wales, Sydney, NSW, Australia; ^5^St. George and Sutherland Clinical School, University of New South Wales, Sydney, NSW, Australia; ^6^George Institute for Global Health, University of New South Wales, Newtown, NSW, Australia

**Keywords:** pregnancy, infancy, microbiome, preeclampsia, hypertensive pregnancy, postpartum

## Abstract

**Objective/Hypothesis:**

To explore potential differences in faecal microbiome between women, and their infants, who had normotensive pregnancies (NP) and those who had a hypertensive pregnancy (HP), either gestational hypertension (GH) or preeclampsia (PE).

**Methods:**

This is a sub study of P4 (Postpartum Physiology, Psychology, and Paediatrics Study) and includes 18 mother-infant pairs: 10 NP and 8 HP (HP as defined by blood pressure > 140/90mmHg; of which 6 had PE, and 2 GH), six months postpartum. The participating mothers collected stool samples from themselves and their infants. 16S rRNA V3-V4 amplicons were used to study the faecal microbiome.

**Results:**

The sample of women and their infants were mostly primiparous (*n* =16) with vaginal birth (*n* = 14). At the time of faecal sampling 8 women were using hormonal contraception, and one HP woman remained on an antihypertensive. All women had blood pressure < 130/80mmHg, and 10 had high BMI (> 30). All infants had started solids, 8 were exclusively breastfed, 1 exclusively formula fed and 9 both. Three infants had been exposed to a course of antibiotics. Six months postpartum, there were no significant differences in alpha or beta diversity between the gut microbiota of HP and NP women (*P* > 0.05). However, a statistically significant difference was detected in alpha diversity between infants following HP and NP, with lower diversity levels in HP infants (*P* < 0.05). It was also found that at a genus and species level, the gut microbiota of HP women was enriched with *Bifidobacterium* and *Bifidobacterium* sp. and depleted in *Barnesiella* and *Barnesiella intestinihominis* when compared to NP women (*P* < 0.05). Similarly, the gut microbiota of infants born from HP was enriched in *Streptococcus infantis* and depleted in *Sutterella*, *Sutterella* sp., *Bacteroides* sp. and *Clostridium aldenense* compared to infants born from NP (*P* < 0.05).

**Discussion:**

While our findings are at best preliminary, due to the very small sample size, they do suggest that the presence of hypertension in pregnancy may adversely affect the maternal microbiota postpartum, and that of their infants. Further analysis of postpartum microbiome data from future studies will be important to validate these early findings and provide further evidence about the changes in the microbiota in the offspring of women following hypertensive disorders of pregnancy (HDP), including possible links to the causes of long-term cardiovascular disease, the prevalence of which is increased in women who have experienced HDP.

## Introduction

There is mounting evidence of the impact of the human microbiome on health, including during pregnancy. In pregnancy, gut dysbiosis has been associated with several important pregnancy complications including gestational diabetes (GDM) (Crusell et al., 2018; Wang et al., 2018; Tenenbaum-Gavish et al., 2020) and hypertensive disorders of pregnancy (HDP) (Chen et al., 2020). HDP affect 5-10% of pregnant women globally (Duley, 2009), with the most common HDP being preeclampsia (PE) and gestational hypertension (GH). PE is a multisystem disorder associated with both severe maternal effects (including renal, neurological, hepatic, haemotologic), and fetal complications including fetal growth restriction, placental abruption and prematurity (Brown Mark et al., 2018). GH (hypertension in pregnancy without the multisystem features characterising PE) has minimal short-term pregnancy impacts but progresses to PE in 25-50% of cases and in the long term, women with PE or GH are at increased risk of cardiovascular disease related morbidity and mortality, with this risk apparent within 10 years of an affected pregnancy and continuing lifelong (Theilen et al., 2016; Wu et al., 2017; Arnott et al., 2020).

The aetiology of HDP, in particular preeclampsia, remains unclear, though it appears to be multifactorial, which raises the possibility of under-studied factors such as the maternal microbiome. These include *Faecalibacterium* sp. which is involved in the metabolism of dietary fibre and providing anti-inflammatory effects, and *Akkermansia* sp. which have actions in strengthening intestinal barrier functions in the gut. Chen et al. found *Faecalibacterium* and *Akkermansia* species to be depleted in the faecal microbiome of women with PE (Chen et al., 2020). Lv et al. demonstrated that disruption in gut microbiota in women with PE persisted at 6 weeks postpartum (Lv et al., 2019).

Another field of uncertainty is the long-term effects of HDP on the fetus and child development. Children of pregnancies complicated by preeclampsia have increased blood pressure and body mass index (Davis et al., 2012). Several studies have examined the gut microbiota during pregnancy in recent years (Koren et al., 2012; Aagaard et al., 2012; Aagaard et al., 2014; Nuriel-Ohayon et al., 2019; van der Giessen et al., 2020), but few have examined the postpartum stage in both mother and infant. Further analysis of the infant microbiota may help to inform our understanding of how maternal factors can influence infant health. As such, it is pertinent to clarify the pathophysiological basis of long-term cardiovascular effects of HDP and establish biomarkers which may aid in the prediction of poor outcomes.

The aim of this study was to explore possible differences in faecal microbiota six months postpartum in women and their infants who had normotensive pregnancies (NP) versus those who had gestational hypertension or preeclampsia (HP). We hypothesised that the faecal microbiota of infants born to mothers with HP are altered from those born to NP mothers.

## Materials and Methods

This is a sub-study of a large prospective cohort study known as P4 (Postpartum Physiology, Psychology and Paediatrics) being conducted at St George Hospital which is a metropolitan teaching hospital that serves a diverse sociodemographic patient population in Sydney. The P4 Study aims to investigate maternal health (physical and psychological) and child health (physical and developmental) in the first 5 years after HP (PE and GH) compared to NP. A detailed P4 study protocol has been published (Davis et al., 2016) as has six months postpartum maternal P4 data which found higher blood pressure and more adverse cardiometabolic health markers in women after PE versus NP (Brown Mark et al., 2020).

Following an ethics amendment (ethical approval from South-Eastern Sydney Local Health District Human Research Ethics Committee, reference number: 12/195), mothers from the P4 study were invited to participate in this microbiome sub-study at 6 months postpartum. Women who were due for their six-month P4 follow up visit were contacted with information about the sub-study and provided their written informed consent to participate. Inclusion criteria for this sub-study, in addition to P4 inclusion criteria, were: no maternal antibiotic usage for the three months prior to the visit; no maternal probiotic usage within one month prior to their 6 month P4 follow up visit; and no history of an acute diarrheal illness for the month prior to and at the time of visit.

### Sample Collection and DNA Extraction

Stool samples were collected from the participating mothers and infants through self-collection. Samples were aliquoted and stored at -80°C and extracted using the PSP Spin Stool DNA extraction kit (Invitek) with additional mechanical lysis for 5 minutes at 30 Hz, using the Tissuelyzer II (Qiagen) to ensure complete lysis of bacterial cells. DNA concentrations were then quantified (Qubit) and the DNA subjected to 16S rRNA V3-V4 amplicon sequencing on the Illumina Miseq platform. QIIME 2 was used to process and perform quality control on sequencing data. Chimeric and primer sequences were removed using DADA2. Sequence alignment and taxonomic classification was performed as per previously published methodology (Bokulich et al., 2018). The dataset used for analysis is available in full at NCBI repository under BioProject ID PRJNA701500.

### Bioinformatic Analysis

The 16S rRNA gene forward and reverse reads were imported into Qiime2 (Bolyen et al., 2019). The DADA2 pipeline (Callahan et al., 2016) was used for detecting and correcting Illumina amplicon sequences, removal of primers and chimeric reads, and assembly into sequence variants (SV)/operational taxonomic units (OTUs) (Callahan et al., 2017). Taxonomy was assigned using a naïve Bayes classifier trained on the Greengenes database13_8. Alpha-diversity metrics investigated included Faith’s phylogenetic diversity (PD), Pielou’s evenness, Observed operational taxonomy units (OTUs) and Shannon’s diversity index which were calculated using qiime2-q2-diversity. Beta-diversity metrics was calculated using qiime2 and distance metrics were quantified using Bray–Curtis dissimilarity index. Statistical analysis was conducted using R v3.6.3 (R Core Team, 2020) in RStudio v1.3.959 (RStudio Team, 2020). Data was visualized using principal coordinates analysis (PCoA) plots and alpha diversity plots generated within RStudio using ggplot2 (Wickham, 2016) and phyloseq (McMurdie and Holmes, 2013). Other packages used included dplyr (Wickham et al., 2019) and qiime2R (Bisanz et al., 2018).

The Wilcoxon rank sum test was used for alpha diversity comparisons between two groups, and the Kruskal–Wallis test, with Dunn’s *post-hoc* test, was used for alpha diversity comparisons between three groups. Distance based permutation multivariate analysis of variance (PERMANOVA) (Koleva et al., 2015) was performed to test the null hypothesis that there were no differences in microbial community structure across treatments at a significance level of *P* = 0.05 based on 999 permutations. *P* values for alpha and beta diversity measures were false discovery rate (FDR) corrected using the Benjamini*-*Hochberg procedure, with 0.05 as the significance threshold.

Linear discriminant analysis Effect Size (LEfSe) (Segata et al., 2011) was used to detect differences in taxonomic abundance between groups. Taxa were identified as differentially abundant through Kruskal-Wallis testing on classes and Wilcoxon rank-sum pairwise testing on subclasses. This was combined with a linear discriminant analysis (LDA) model to evaluate effect size. Using LEfSe, taxa were considered significantly differentially abundant if their *P* value was < 0.05 and their LDA log score was > 2. FDR corrections were additionally performed using the Benjamini*-*Hochberg procedure, with 0.05 as the significance threshold.

## Results

### Maternal and Infant Characteristics

Ten NP and 8 HP (6 preeclampsia, 2 gestational hypertension) mother-infant pairs participated in the sub study. Maternal characteristics are shown in **Table 1**, with women predominantly primiparous (*n* = 16), with vaginal birth (*n* = 14). One HP woman had a history of beta-thalassaemia minor and another HP woman developed cholestasis of pregnancy in addition to PE. Two HP women had high BMI (> 30 kg/m^2^). None of the women in either group had pre-existing or gestational diabetes, thyroid disease, or a history of gastrointestinal disease. Eight women reported pre-pregnancy alcohol consumption, all ceased during pregnancy and 5 recommenced postpartum (average of 3-5 standard drinks/week at time of six-month postpartum sampling). One HP woman was vegetarian, otherwise all other women reported no special dietary requirements. A statistically significant difference in diastolic blood pressure and mode of birth was found between the two groups of mothers involved in the sub-study.

**Table 1:** Maternal characteristics.

	Total Cohort (*n* = 18)	Normotensive Pregnancy (NP) (*n* = 10)	HP = Preeclampsia (PE) or Gestational Hypertension (GH) (*n* = 8)	*p* value NP vs HP
	Mean ± SD	Mean ± SD	Mean ± SD	
Age (years) at time of birth	30.8 ± 4.4	30.7 ± 3.9	30.9 ± 5.1	*0.64*
Systolic blood pressure (mmHg)	101.8 ± 8.6	101.5 ± 10.7	102.3 ± 5.7	*0.96*
Diastolic blood pressure	69.8 ± 5.4	67.3 ± 4.8	72.9 ± 4.6	***0.049***
Height (cm)	165.1 ± 8.0	164.1 ± 8.6	166.3 ± 7.6	*0.76*
Weight 6 months postpartum (kg)	70.5 ± 17.3	64.7 ± 10.0	77.8 ± 22.2	*0.22*
Gestational weight gain (kg)	11.1 ± 6.0	11.1 ± 4.9	11.2 ± 8.0	*0.51*
Body Mass Index (BMI) (kg/m2)	25.7 ± 4.9	24.1 ± 3.6	27.8 ± 5.7	*0.19*
	***n* (%)**	***n* (%)**	***n* (%)**	
*Mode of birth:*				
Vaginal birth	14 (77)	10 (100)	4 (50)	***0.029***
Caesarean delivery	4 (22)	0 (0)	4 (50)	
*Smoking:*				*0.32*
Pre-pregnancy	4 (22)	2 (20)	4 (50)	*Pre-pregnancy use*
During pregnancy	0 (0)	0 (0)	0 (0)	
Postpartum	1 (6)	0 (0)	1 (13)	
*Alcohol:*				
Pre-pregnancy	8 (44)	2 (20)	6 (75)	*0.054*
During pregnancy	0 (0)	0 (0)	0 (0)	*Pre-pregnancy use*
Postpartum	5 (28)	2 (20)	3 (38)	
	(mean ± SD)	(mean ± SD)	(mean ± SD)	
Postpartum standard drink per week	3.4 ± 2.3	2 ± 1.4	3.8 ± 2.4	
*Medication use:*				
Multivitamin	6 (33)	4 (40)	2 (25)	*0.64*
Antidepressant	1 (6)	0 (0)	1 (16)	*0.45*
Antihypertensive	1 (6)	0 (0)	1 (16)	*0.45*
Hormonal contraceptive	8 (44)	4 (40)	4 (50)	*1.00*

NP, normotensive pregnancy; HP, hypertensive pregnancy; PE, preeclampsia; GH, gestational hypertension; mmHg, milimetres of mercury; cm, centimetres; kg, kilograms.

Bold refers to those p values that are < 0.05.

Infant characteristics are shown in **Table 2**. All infants born to normotensive mothers (NP) were born vaginally (*n* = 10). Of the hypertensive group, all infants of women in the GH group were born by caesarean delivery (*n* = 2) and two of the six mothers in the PE group had caesarean deliveries. All but one infant (from the NP group) had commenced solid food intake. Three infants had received antibiotics in their lifetime, two at the time of delivery: one, born at 32 weeks and 6 days in the PE group who also used a paediatric multivitamin; the second a term baby born at 39 weeks and 6 days gestation, for suspected chorioamnionitis; and the third had daily cephalexin exposure since birth for a duplex kidney. A statistically significant difference was found in gestational age at birth between the two groups of infants involved in the sub-study.

**Table 2:** Infant characteristics.

	Total Cohort (*n* = 18)	Normotensive Pregnancy (NP) (*n* = 10)	Preeclampsia (PE) Gestational Hypertension (GH) (*n* = 8)	*p* value
	Mean ± SD	Mean ± SD	Mean ± SD	
Birthweight (kg)	3.3 ± 0.5	3.4 ± 0.4	3.2 ± 0.7	*0.20*
Gestation at birth (weeks)	38.6 ± 2.3	39.6 ± 1.3	37.4 ± 2.4	***0.016***
Weight at 6 months (kg)	7.95 ± 1.10	7.76 ± 0.84	8.19 ± 1.38	*0.85*
	***n* (%)**	***n* (%)**	**n (%)**	
*Feeding method:*				
Breastfed	8 (44)	4 (40)	4 (50)	*1.00*
Formula	1 (6)	0 (0)	1 (12)	*0.45*
Mixed	9 (50)	6 (60)	3 (38)	*0.64*
Commenced solids	17 (94)	9 (90)	8 (100)	*1.00*
Antibiotic treatment	3 (17)	1 (10)	2 (25)	*0.56*

Bold refers to those p values that are < 0.05.

### Differences in the Gut Microbiota of Mothers at 6 Months Postpartum and Infants at 6 Months of Age

**Figure 1:**
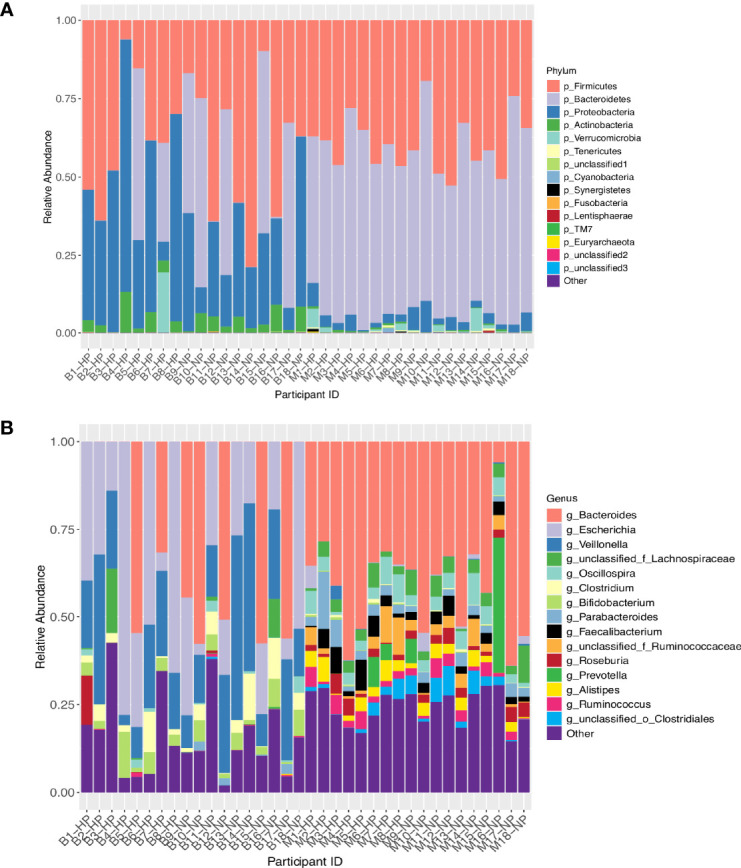
**(A)** Relative abundance of the top 15 bacterial phyla. **(B)** Relative abundance of the top 15 bacterial genera. The X axis represents participants in the study. The Y axis is a scale of relative abundance out of 1. M, mother; B, baby; NP, normotensive pregnancy; HP, hypertensive pregnancy. Taxon levels are abbreviated with p, phylum; c, class; o, order; f, family; g, genus and s, species.

**Figure 2:**
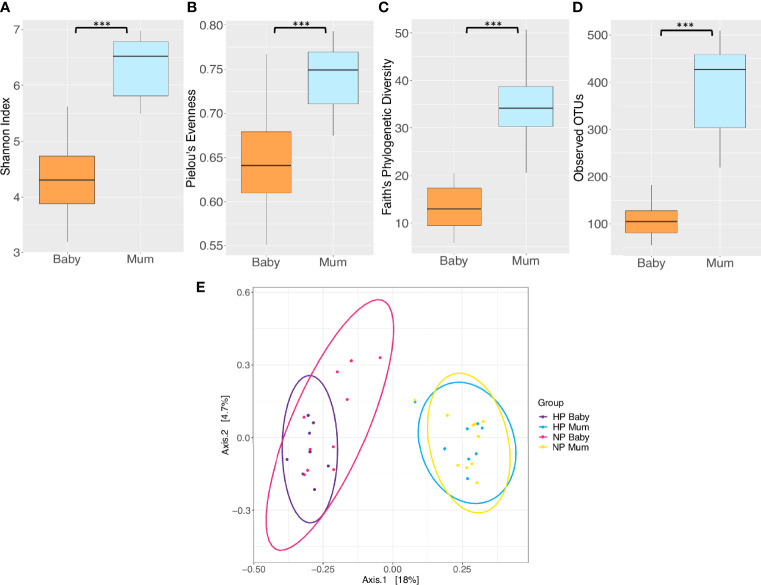
Comparison of microbiota diversity between mothers (*n* = 18) and their babies (*n =* 18) based on faecal samples collected at 6 months postpartum and at six months of age respectively. Alpha diversity measured by **(A)** Shannon index **(B)** Pielou’s evenness **(C)** Faith’s phylogenetic diversity **(D)** Observed operational taxonomic units (OTUs). Boxes represent the interquartile range (IQR), the line inside the box indicates the median and whiskers represent values 1.5 × IQR from the first and third quartiles respectively. Differences between mothers and babies were tested using the Wilcoxon sum rank test and false discovery rate (FDR) corrected. ***P < 0.001. **(E)** Principal Coordinates Analysis (PCoA) of microbiota community structure in mothers and babies. NP, normotensive pregnancy; HP, hypertensive pregnancy. The points represent individual samples from mothers and their babies, and the ellipses illustrates the 95% confidence intervals of multivariate normal distribution.

**Figures 1A, B** illustrate the relative abundance of the top 15 bacterial phyla and genera in the maternal and infant gut microbiota at 6 months postpartum and six months of age, respectively. The maternal and infant gut microbiota were primarily composed of phyla Firmicutes, Bacteroidetes and Proteobacteria. *Bacteroides, Escherichia* and *Veillonella* were the main genera composing the infant gut microbiota whilst *Bacteroides* and *Oscillospira* were the primary genera in the maternal gut microbiota. Overall, mothers had a significantly greater alpha diversity compared to that of infants, when measured by Shannon’s diversity index, Pielou’s evenness, Faith’s phylogenetic diversity (PD) and observed operational taxonomic units (OTUs) (*P* < 0.001) (**Figure 2A–D** and **Supplementary Table 1**). Furthermore, analysis revealed significant differences in beta diversity between mothers and infants, showing a fundamental variation in microbial community composition (*P* = 0.001) (**Supplementary Table 2**). This is illustrated by the separate clustering of maternal and infant samples in the PCoA graph (**Figure 2E**).

### Changes in Gut Microbiota Composition Between Women After HP and NP

**Figure 3:**
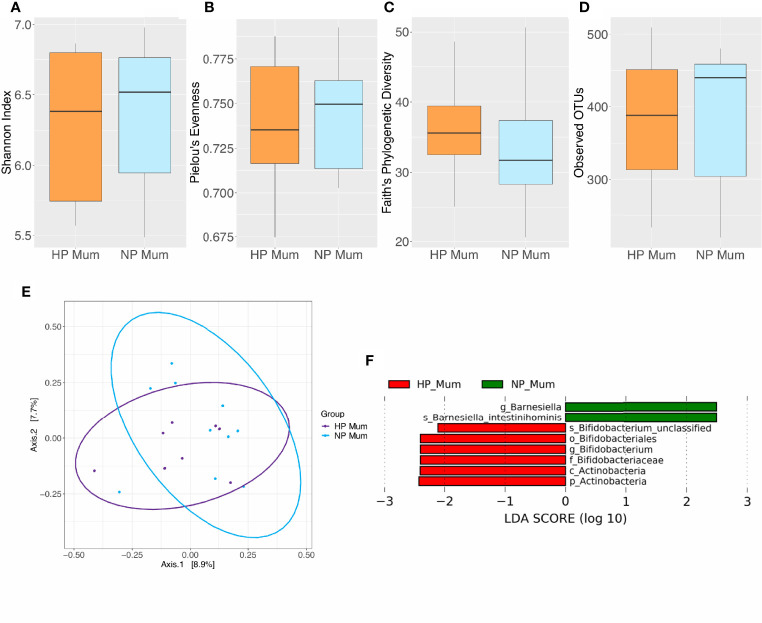
Comparison of microbiota composition and diversity between hypertensive pregnancy (HP) mothers (*n* = 8) and normotensive pregnancy (NP) mothers (*n =* 10) based on faecal samples. Alpha diversity measured by **(A)** Shannon index **(B)** Pielou’s evenness **(C)** Faith’s phylogenetic diversity **(D)** Observed operational taxonomic units (OTUs). Boxes represent the interquartile range (IQR), the line inside the box indicates the median and whiskers represent values 1.5 × IQR from the first and third quartiles respectively. Differences between groups were tested using the Wilcoxon sum rank test and false discovery rate (FDR) corrected. Overall, no significant differences were observed in alpha diversity between HP and NP groups. **(E)** Principal Coordinates Analysis (PCoA) of microbiota community structure in HP and NP mothers. The points represent individual samples from mothers and the ellipse illustrates the 95% confidence intervals of multivariate normal distribution. **(F)** Differentially abundant bacterial taxa as identified by Linear discriminant analysis Effect Size (LEfSe). Bacterial taxa were classified as differentially abundant if their *P* value was < 0.05 and their linear discriminant analysis (LDA) log score was > 2. Taxa enriched in HP mothers are indicated by red, and taxa enriched in NP mothers are indicated by green. Taxon levels are abbreviated with p_ = phylum, c_ = class, o_ = order, f_ = family, g_ = genus and s_ = species. LDA effects sizes and significant *P* values are in detailed in **Supplementary Table 5**.

No significant differences in alpha or beta diversity were detected at 6 months postpartum between HP and NP women (*P* > 0.05) (**Figures 3A–E** and **Supplementary Tables 3, 4**). However, LEfSe analysis revealed differentially abundant taxa at 6 months postpartum between HP and NP women (**Figure 3F** and **Supplementary Table 5**). The gut microbiota of HP women was enriched in phylum Actinobacteria, order Bifidobacteriales, family Bifidobacteriaceae, genus *Bifidobacterium* and species *Bifidobacterium* sp. compared to NP women (LDA > 2, *P* < 0.05). Conversely, the gut microbiota of HP women was depleted in genus *Barnesiella* and species *Barnesiella intestinihominis* when compared to NP women (LDA > 2, *P* < 0.05). These differences in gut microbiota taxonomic composition between HP and NP women did not remain significant after FDR correction (*P* > 0.05). However, taxa identified as significant prior to FDR correction were considered as potential biomarkers for future investigation. This is due to their identification through LEfSe’s rigorous multistep process of biomarker discovery and the acknowledgement that future investigation with larger sample sizes may detect significance.

### Impact of Clinical Factors on the Maternal Gut Microbiota

**Figure 4:**
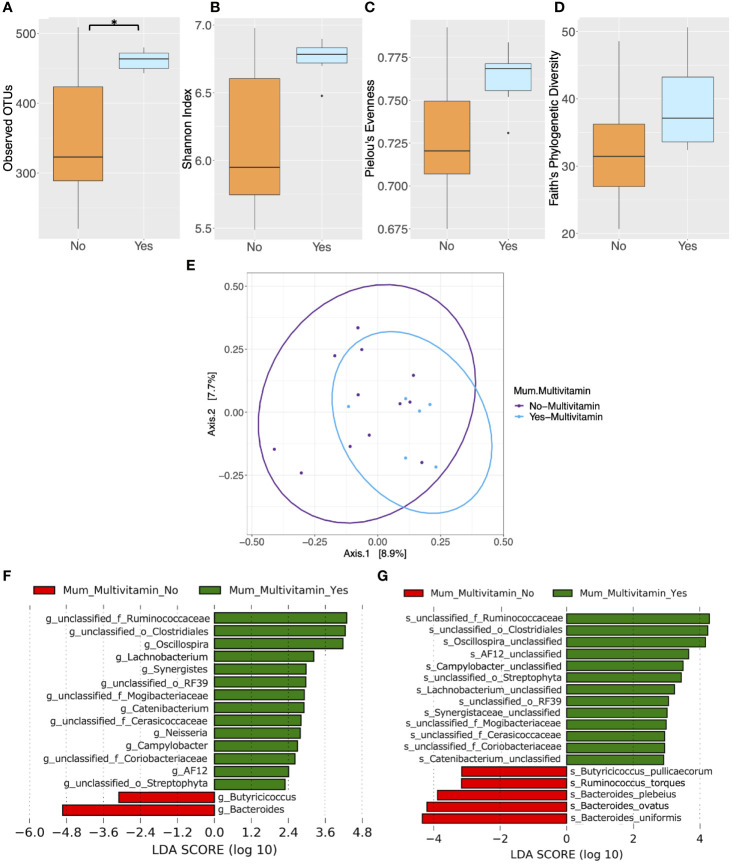
Comparison of microbiota composition and diversity between mothers who took multivitamins (*n* = 6) and those who did not (*n* = 12) based on faecal samples collected at 6 months postpartum. Alpha diversity measured by **(A)** Observed operational taxonomic units (OTUs) **(B)** Shannon index **(C)** Pielou’s evenness **(D)** Faith’s phylogenetic diversity. Yes = mother took multivitamins, No = mother did not take multivitamins. Boxes represent the interquartile range (IQR), the line inside the box indicates the median and whiskers represent values 1.5 × IQR from the first and third quartiles respectively. Differences between groups were tested using the Wilcoxon sum rank test and false discovery rate (FDR) corrected. *P < 0.05 **(E)** Principal Coordinates Analysis (PCoA) of microbiota community structure based on maternal multivitamin intake. The points represent individual samples from mothers and the ellipse illustrates the 95% confidence intervals of multivariate normal distribution. **(F)** Differentially abundant bacterial genera as identified by Linear discriminant analysis Effect Size (LEfSe). **(G)** Differentially abundant bacterial species as identified by LEfSe. Bacterial taxa were classified as differentially abundant if their *P* value was < 0.05 and their linear discriminant analysis (LDA) log score was > 2. Taxa enriched in mothers who did not take multivitamins are indicated by red, and taxa enriched in those who did take multivitamins are indicated by green. Taxon levels are abbreviated with p, phylum; c, class; o, order; f, family; g, genus and s, species. LDA effects sizes and significant *P* values are in **Supplementary Tables 10–11**.

Several other maternal characteristics with the potential to influence microbial composition were analyzed. Pre-pregnancy and postpartum smoking status and alcohol consumption were found to not be associated with differences in microbial alpha and beta diversity in mothers (*P* > 0.05) (**Supplementary Tables 6, 7, 9**). However, pregnancy and postpartum multivitamin use was associated with elevated alpha diversity as measured by observed OTUs in the maternal gut microbiota (*P* = 0.040) (**Figure 4A**, **Supplementary Table 8**). Mean Shannon’s index, Pielou’s evenness and Faith’s PD measures were higher in mothers who took multivitamins, although not significantly (*P* > 0.05) (**Figures 4B–D** and **Supplementary Table 8**). However, significant differences in beta diversity were found between women grouped according to multivitamin intake (*P* = 0.035) (**Figure 4E** and **Supplementary Table 9**). Additionally, LEfSe analysis revealed that multivitamin intake had a significant impact on gut microbiota taxonomic composition in maternal cohorts at the genus (**Figure 4F** and **Supplementary Table 10**) and species level (LDA > 2, *P* < 0.05) (**Figure 4G** and **Supplementary Table 11**). These differences in microbiota composition based on multivitamin intake did not remain significant following FDR correction (*P* > 0.05).

### Changes in Gut Microbiota Diversity and Composition Between Infants Born From HP and NP

**Figure 5:**
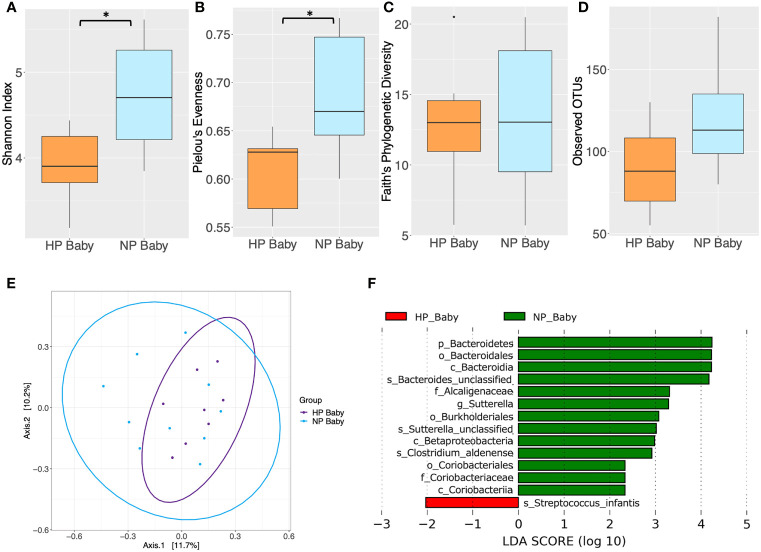
Comparison of diversity indices between hypertensive pregnancy (HP) infants (*n* = 8) and normotensive pregnancy (NP) infants (*n =* 10) based on faecal samples collected at 6 months of age. Alpha diversity measured by **(A)** Shannon index **(B)** Pielou’s evenness **(C)** Faith’s phylogenetic diversity **(D)** Observed operational taxonomic units (OTUs). Boxes represent the interquartile range (IQR), the line inside the box indicates the median and whiskers represent values 1.5 × IQR from the first and third quartiles respectively. Differences between mothers and babies were tested using the Wilcoxon sum rank test and false discovery rate (FDR) corrected. *P < 0.05. **(E)** Principal Coordinates Analysis (PCoA) of microbiota community structure in HP babies and NP babies. The points represent individual samples from babies and the ellipse illustrates the 95% confidence intervals of multivariate normal distribution. **(F)** Differentially abundant bacterial taxa as identified by Linear discriminant analysis Effect Size (LEfSe). Bacterial taxa were classified as differentially abundant if their *P* value was < 0.05 and their linear discriminant analysis (LDA) log score was > 2. Taxa enriched in the HP babies are indicated by red, and taxa enriched in the NP babies are indicated by green. Taxon levels are abbreviated with p_ = phylum, c_ = class, o_ = order, f_ = family, g_ = genus and s_ = species. LDA effects sizes and significant *P* values are in **Supplementary Table 14**.

Significant differences in alpha diversity were found between HP and NP infants. Both Shannon’s diversity (*P* = 0.031) and Pielou’s evenness (*P* = 0.031) indices were significantly lower in infants born to HP women compared to those born to NP women (**Figures 5A, B** and **Supplementary Table 12**). Similarly, Faith’s PD and the mean number of observed OTU’s were lower, although not significantly, in infants whose mothers had HP compared to those who had NP (**Figures 5C, D** and **Supplementary Table 12**). However, there were no significant difference in beta diversity between infants born from HP or NP women (*P* > 0.05) (**Figure 5E** and **Supplementary Table 13**). LEfSe analysis revealed taxonomic differences in the gut microbiota of HP versus NP infants (**Figure 5F** and **Supplementary Table 14**). The gut microbiota of HP infants was depleted in phylum Bacteroidetes, classes Betaproteobacteria, Coriobacteriia and Bacteroidia, orders Bacteroidales, Burkholderiales and Coriobacteriales, families Alcaligenaceae and Coriobacteriaceae, genus *Sutterella* and species *Clostridium aldenense, Sutterella* sp. and *Bacteroides* sp., compared NP infants (LDA > 2, *P* < 0.05). Conversely the species *Streptococcus infantis* was enriched in HP compared to NP infants (LDA > 2, *P* < 0.05). Differences in taxonomic composition between NP and HP infants did not remain significant after FDR correction (P > 0.05). However, in this preliminary study, taxa identified as significant by LEfSe prior to FDR correction, were explored as potential biomarkers for further investigation in larger future studies.

### Impact of Clinical Factors on the Infant Gut Microbiota

**Figure 6:**
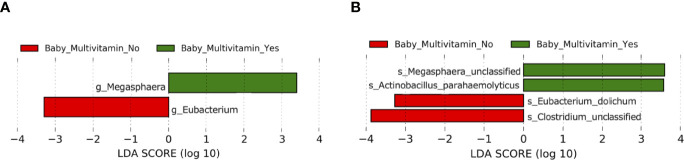
Comparison of taxonomic composition between babies with mothers who took multivitamins in gestation (*n* = 6) and babies with mothers who did not take multivitamins in gestation (*n* = 12). **(A)** Differentially abundant bacterial genera as identified by Linear discriminant analysis Effect Size (LEfSe). **(B)** Differentially abundant bacterial species as identified by LEfSe. Bacterial taxa were classified as differentially abundant if their *P* value was < 0.05 and their linear discriminant analysis (LDA) log score was > 2. Taxa enriched in babies whose mothers did not take multivitamins in gestation are indicated by red, and taxa enriched in babies whose mothers did take multivitamins in gestation are indicated by green. Taxon levels are abbreviated with p_ = phylum, c_ = class, o_ = order, f_ = family, g_ = genus and s_ = species. LDA effects sizes and significant *P* values are in **Supplementary Tables 21–22**.

In infants, maternal pre-pregnancy smoking status, pre-pregnancy alcohol consumption, birthing methods (caesarian or vaginal delivery), feeding methods (breast, bottle and mixed feeding) and pregnancy multivitamin intake had no significant effect on alpha and beta diversity indices (*P* > 0.05) (**Supplementary Figure 1** and **Supplementary Tables 15–20**). However, alpha diversity measures were higher, albeit non-significantly so, in babies whose mothers took multivitamins during pregnancy. LEfSe analysis identified differentially abundant taxa in the gut microbiota of babies when grouped according to maternal multivitamin intake at both genus (**Figure 6A** and **Supplementary Table 21**) and species level (LDA > 2, *P* < 0.05) (**Figure 6B** and **Supplementary Table 22**). These differences in infant microbiota composition based on maternal multivitamin intake did not remain significant following FDR correction, and this may be in part due to the small sample size of the study (*P* > 0.05).

## Discussion

This paper uniquely compares the microbiome six months postpartum of mothers and their infants with and without a hypertensive disorder of pregnancy. Understanding the long-term impacts of hypertensive disorders of pregnancy on mothers and infants is crucial, especially as early interventions to improve cardiovascular and metabolic health outcomes are being made possible through epidemiological, physiological and biochemical studies (Theilen et al., 2016; Wu et al., 2017; Brown Mark et al., 2018; Lui et al., 2019).

This study forms part of the larger P4 study investigating the long term impacts of HDP. The main findings to date have been (a) in mothers, women six months after HP versus NP show more markers of cardiovascular and metabolic disease susceptibility including; higher blood pressure, higher fat mass, more tendency to insulin resistance, and higher rates of micronutrient insufficiency (b) in infants, more infants were born small for gestational age (SGA) after HP, and SGA infants, regardless of hypertensive pregnancy status, were more likely to experience rapid weight gain 0-6 months (rapid catch-up growth being itself associated with future cardiovascular disease) (Brown Mark et al., 2020; Gow et al., 2021; McLennan et al., 2021). One potential mechanism for these observations could be microbiota related. The findings of our study when viewed mechanistically allow for further evaluation of the potential physiological reasons behind our findings and illuminate further areas for scientific exploration.

When conducting the analysis for this paper, using 16S rRNA sequencing we looked into the individual bacteria that resulted to try and postulate links and associations with the underlying maternal and infant physiology following both NP and HP.

Differences were observed in mothers and infants based on whether the pregnancy was normotensive or hypertensive. Maternally, in NP there was an increase in *Barnesiella* and *Barnesiella intestinihominis* compared to HP. Fielding et al. found that the passage of *Barnesiella intestinihominis* sp. via faecal microbial transplantation (FMT) into germ-free mice led to increased muscle mass and strength (Fielding et al., 2019). By extrapolation to humans, it could be argued that these bacterial species are beneficial in normotensive women.

The faecal samples of HP mothers were found to be enriched with six types of Actinobacteria, sharing down to the genus *Bifidobacterium.* This finding is biologically plausible given that mother to infant transmission of intestinal bacteria has been observed (Makino et al., 2013), and the presence of *Bifidobacterium* sp. in the gut of infants aids the digestion of human milk oligosaccharides (HMOs) (Marcobal and Sonnenburg, 2012; Underwood et al., 2017). Considering that HDP, particularly preeclampsia and its associated placental dysfunction can lead to fetal growth restriction and the birth of small neonates, an increase in *Bifidobacterium*, would seemingly only aid the energy and nutrient extraction from maternal breastmilk in order to reach maximal infant growth velocities once born. Venagas et al. (Parada Venegas et al., 2019) suggest that the presence of butyrate producing Actinobacteria, and acetate and propionate producing *Bifidobacterium*, may also play a role in hypertension. In the current study, the enrichment of phylum Actinobacteria and order Bifidobacteriales found in HP women, and the statistically significant difference in maternal diastolic blood pressure between NP and HP at 6 months postpartum, suggest that the butyrate and proportionate producing microbiota is potentially correlated to elevated blood pressure (Gomez-Arango et al., 2016; Chen et al., 2020).

In this study, one enriched species of bacteria from the Firmicutes phylum, *Streptococcus infantis* was found in the infants born from HP mothers. This bacterium is identified in scientific literature under the Mitis group of commensals recovered from upper respiratory tract specimens (Pimenta et al., 2019), however, its role in infant faeces remains unclear. The current study found a reduction in *Sutterella* sp. in the infants born from HP mothers. Wang et al. found increased *Sutterella* species in children who went on to develop autism spectrum disorder (Wang et al., 2013) and a meta-analysis performed by Maher et al. showed a 35% increase in the odds of having a child with autism in hypertensive disorder exposed pregnancies (Maher et al., 2018). These studies results differ from the current findings, which only serve to demonstrate the multifactorial nature of complex diagnoses, such as autism spectrum disorder, and the importance of long term follow up of the infants born from pregnancies with complications.

Infants born to HP mothers were found to be depleted in phylum Bacteroidetes and Bacteroides sp. which are generally viewed as gastrointestinal tract commensals beneficial to human functioning. There are a number of perinatal and postnatal factors associated with reduced Bacteroidetes or *Bacteroides* abundance in infant stool including caesarean delivery (Bäckhed et al., 2015; Vatanen et al., 2016; Bokulich et al., 2016), exclusive breastfeeding (Penders et al., 2006; Fallani et al., 2010) and maternal high fat diet (Chu et al., 2016). HP infants in this study were more likely to be born by caesarean, so their Bacteroidetes and *Bacteroides* sp. depletion may not be solely attributable to their HP exposure, nonetheless, the findings indicates a less beneficial infant microbial ecosystem after HP. Additionally, whilst the overall relative abundance of phylum Bacteroidetes was lower in HP compared to NP infants, at an individual level several NP infants also exhibited low levels of Bacteroidetes, suggesting the potential of multifactorial influences upon its abundance.

Whilst multivitamin intake in the mother did not change infant faecal microbiota beta diversity, there was a trend towards alpha diversity measures being higher, albeit non significantly so, in babies whose mothers took multivitamins during pregnancy. Of note, differentially abundant taxa at both genus and species level, were present prior to FDR adjustment, in infants grouped by maternal multivitamin intake. Of note, the larger P4 study showed that HP and non-breastfeeding status were associated with maternal micronutrient insufficiency (Siritharan et al., 2021).

Chu et al. performed the largest study of whole metagenomic sequencing on neonatal and infant stool to date, and demonstrated that by six weeks of age the infant microbial community structure and function had significantly expanded and diversified (Chu et al., 2016). The design of the current study, testing at a single time point of 6 months postpartum, was not able to detect initial fluctuations of the microbiota in infants included. In HP infants faeces, Coriobacteriia were also reduced. It is postulated that Coriobacteriaceae carry out important functions such as the conversion of bile salts and steroids, as well as the activation of dietary polyphenols (Clavel et al., 2014), whilst also playing a role in vitamin K2 production (Clavel et al., 2014). Liu et al. also demonstrated that an increase in Coriobacteriaceae within Actinobacteria might contribute to improved glucose tolerance and insulin sensitivity in diabetic animal models (Liu et al., 2018). Extrapolating this, it is biologically plausible that the offspring born to NP mothers have an increased abundance of this bacterium. Infants born to HP mothers do not, and this could potentially be related to longer term health outcomes, given the known risk of obesity and elevated body mass index in offspring born to mothers with preeclampsia (Davis et al., 2012).

### Strengths and Limitations

Study limitations include the small sample size of this pilot/proof of concept study, and diverse characteristics within the sample, as well as any bias inherent in the group of women who chose to participate in this sub-study. Another limitation is the lack of microbiome samples from mothers in late pregnancy or at the time of birth. Strengths include that this is the first Australian examination of mothers and children after HP versus NP, and, as a pilot study, provides proof of acceptability and feasibility for the larger cohort of the Microbiome in Maternity Study (MUMS) (Susic et al., 2020).

A comprehensive Australian based maternity microbiome study (MUMS) will delve more deeply into the questions raised, including impacts on the infants born from hypertensive pregnancies. These questions and scientific exploration will then naturally lead to proposing whether supplementation of specific microbial taxa could play a role in ameliorating the long-term impact of hypertensive disorders during pregnancy on the next generation.

## Conclusion

The pathophysiology of hypertensive pregnancies and the impact on the next generation is a complex and difficult subject to study. There are multiple confounding factors that impact the interpretation of results from emerging microbiome data. We have shown that microbes may indeed be involved in the transgenerational impact of hypertensive pregnancies, through observing changes in the gut microbiota of both women who experienced hypertension during pregnancy and their offspring compared to normotensive groups. Further longitudinal studies delving deeper into this area are warranted given the potential for future preventative and therapeutic considerations.

## Data Availability Statement

The datasets presented in this study can be found in online repositories. The names of the repository/repositories and accession number(s) can be found below: BioProject ID PRJNA701500.

## Ethics Statement

The studies involving human participants were reviewed and approved by South-Eastern Sydney Local Health District Human Research Ethics Committee, reference number: 12/195. Written informed consent to participate in this study was provided by the participants’ legal guardian/next of kin.

## Author Contributions

DS, LR, GD, EE-O, and AH contributed to conception and design of the sub-study. AG contributed to the laboratory processing of samples. LW, EM, and XJ have contributed to the bioinformatic analysis. DS, LW, LR, MB, AG, EM, XJ, GD, EE-O and AH have contributed to the editing of the manuscript. All authors contributed to the article and approved the submitted version.

## Funding

This research was funded by the Microbiome Research Centre, University of New South Wales, Sydney, Australia.

## Conflict of Interest

The authors declare that the research was conducted in the absence of any commercial or financial relationships that could be construed as a potential conflict of interest.

## Publisher’s Note

All claims expressed in this article are solely those of the authors and do not necessarily represent those of their affiliated organizations, or those of the publisher, the editors and the reviewers. Any product that may be evaluated in this article, or claim that may be made by its manufacturer, is not guaranteed or endorsed by the publisher.

## References

[B1] Aagaard K. Riehle K. Ma J. Segata N. Mistretta T-A. Coarfa C.. (2012). A Metagenomic Approach to Characterization of the Vaginal Microbiome Signature in Pregnancy. PloS One 7 (6), e36466. doi: 10.1371/journal.pone.0036466 22719832 PMC3374618

[B2] Aagaard K. Ma J. Antony K. M. Ganu R. Petrosino J. Versalovic J.. (2014). The Placenta Harbors a Unique Microbiome. Sci. Trans. Med. 6 (237), 237ra65–237ra65. doi: 10.1126/scitranslmed.3008599 PMC4929217 24848255

[B3] Arnott C. Nelson M. Ramirez M. A. Hyett J. Gale M. Henry A.. (2020). Maternal Cardiovascular Risk After Hypertensive Disorder of Pregnancy. Heart 106 (24), 1927. doi: 10.1136/heartjnl-2020-316541 32404402

[B4] Bäckhed F. Roswall J. Peng Y. Feng Q. Jia H. Kovatcheva-Datchary P.. (2015). Dynamics and Stabilization of the Human Gut Microbiome During the First Year of Life. Cell Host Microbe 17 (5), 690–703. doi: 10.1016/j.chom.2015.04.004 25974306

[B5] Bisanz J. E. Soto-Perez P. Lam K. N. Bess E. N. Haiser J. J. Allen-Vecoe E.. (2018). Illuminating the Microbiome’s Dark Matter: A Functional Genomic Toolkit for the Study of Human Gut Actinobacteria. bioRxiv 304840. doi: 10.1101/304840

[B6] Bokulich N. A. Chung J. Battaglia T. Henderson N. Jay M. Li H.. (2016). Antibiotics, Birth Mode, and Diet Shape Microbiome Maturation During Early Life. Sci. Trans. Med. 8 (343), 343ra82. doi: 10.1126/scitranslmed.aad7121 PMC5308924 27306664

[B7] Bokulich N. A. Kaehler B. D. Rideout J. R. Dillon M. Bolyen E. Knight R.. (2018). Optimizing Taxonomic Classification of Marker-Gene Amplicon Sequences With QIIME 2’s Q2-Feature-Classifier Plugin. Microbiome 6 (1), 90. doi: 10.1186/s40168-018-0470-z 29773078 PMC5956843

[B8] Bolyen E. Rideout J. R. Dillon M. R. Bokulich N. A. Abnet C. C. Al-Ghalith G. A.. (2019). Reproducible, Interactive, Scalable and Extensible Microbiome Data Science Using QIIME 2. Nat. Biotechnol. 37 (8), 852–857. doi: 10.1038/s41587-019-0209-9 31341288 PMC7015180

[B9] Brown M. A. Magee L. A. Kenny L. C. Karumanchi S. A. McCarthy F. P. Saito S.. (2018). Hypertensive Disorders of Pregnancy. Hypertension 72 (1), 24–43. doi: 10.1161/HYPERTENSIONAHA.117.10803 29899139

[B10] Brown M. A. Roberts L. Hoffman A. Henry A. Mangos G. O’Sullivan A.. (2020). Recognizing Cardiovascular Risk After Preeclampsia: The P4 Study. J. Am. Heart Assoc. 9 (22), e018604. doi: 10.1161/JAHA.120.018604 33170079 PMC7763721

[B11] Callahan B. J. McMurdie P. J. Rosen M. J. Han A. W. Johnson A. J. Holmes S. P.. (2016). DADA2: High-Resolution Sample Inference From Illumina Amplicon Data. Nat. Methods 13 (7), 581–583. doi: 10.1038/nmeth.3869 27214047 PMC4927377

[B12] Callahan B. J. McMurdie P. J. Holmes S. P. (2017). Exact Sequence Variants Should Replace Operational Taxonomic Units in Marker-Gene Data Analysis. ISME J. 11 (12), 2639–2643. doi: 10.1038/ismej.2017.119 28731476 PMC5702726

[B13] Chen X. Li P. Liu M. Zheng H. He Y. Chen M.-X.. (2020). Gut Dysbiosis Induces the Development of Pre-Eclampsia Through Bacterial Translocation. Gut 69 (3), 513–522. doi: 10.1136/gutjnl-2019-319101 31900289

[B14] Chu D. M. Antony K. M. Ma J. Prince A. L. Showalter L. Moller M.. (2016). The Early Infant Gut Microbiome Varies in Association With a Maternal High-Fat Diet. Genome Med. 8 (1), 77. doi: 10.1186/s13073-016-0330-z 27503374 PMC4977686

[B15] Clavel T. Lepage P. Charrier C. (2014). “The Family Coriobacteriaceae,” in The Prokaryotes. Eds. Rosenberg E DeLong E. F. Lory S. Stackebrandt E. Thompson F. (Berlin, Heidelberg: Springer). doi: 10.1007/978-3-642-30138-4_343

[B16] Crusell M. K. W. Hansen T. H. Nielsen T. Allin K. H. Rühlemann M. C. Damm P.. (2018). Gestational Diabetes Is Associated With Change in the Gut Microbiota Composition in Third Trimester of Pregnancy and Postpartum. Microbiome 6 (1), 89–89. doi: 10.1186/s40168-018-0472-x 29764499 PMC5952429

[B17] Davis E. F. Lazdam M. Lewandowski A. J. Worton S. A. Kelly B. Kenworthy Y.. (2012). Cardiovascular Risk Factors in Children and Young Adults Born to Preeclamptic Pregnancies: A Systematic Review. Pediatrics 129 (6), e1552. doi: 10.1542/peds.2011-3093 22614768

[B18] Davis G. K. Roberts L. Mangos G. Henry A. Pettit F. O'Sullivan A.. (2016). Postpartum Physiology, Psychology and Paediatric Follow Up Study (P4 Study) - Study Protocol. Pregnancy Hypertens. 6 (4), 374–379. doi: 10.1016/j.preghy.2016.08.241 27939485

[B19] Duley L. (2009). The Global Impact of Pre-Eclampsia and Eclampsia. Semin. Perinatol. 33 (3), 130–137. doi: 10.1053/j.semperi.2009.02.010 19464502

[B20] Fallani M. Young D. Scott J. Norin E. Amarri S. Adam R.. (2010). Intestinal Microbiota of 6-Week-Old Infants Across Europe: Geographic Influence Beyond Delivery Mode, Breast-Feeding, and Antibiotics. J. Pediatr. Gastroenterol. Nutr. 51 (1), 77–84. doi: 10.1097/MPG.0b013e3181d1b11e 20479681

[B21] Fielding R. A. Reeves A. R. Jasuja R. Liu C. Barrett B. B. Lustgarten M. S.. (2019). Muscle Strength Is Increased in Mice That Are Colonized With Microbiota From High-Functioning Older Adults. Exp. Gerontol. 127, 110722–110722. doi: 10.1016/j.exger.2019.110722 31493521 PMC6823114

[B22] Gomez-Arango L. F. Barrett H. L. McIntyre H.D. Callaway L. K. Morrison M. Nitert M. D.. (2016). Increased Systolic and Diastolic Blood Pressure Is Associated With Altered Gut Microbiota Composition and Butyrate Production in Early Pregnancy. Hypertension 68 (4), 974–981. doi: 10.1161/HYPERTENSIONAHA.116.07910 27528065

[B23] Gow M. L. Roberts L. M. Henry A. Davis G. Mangos G. Pettit F.. (2021). Growth From Birth to 6-Months of Infants With and Without Intrauterine Preeclampsia Exposure. J. Dev. Orig. Health Dis. doi: 10.1017/S2040174421000167 33977898

[B24] Koleva P. T. Kim J.-S. Scott J. A. Kozyrskyj A. L.. (2015). Microbial Programming of Health and Disease Starts During Fetal Life. Birth Defects Res. C Embryo Today 105 (4), 265–277. doi: 10.1002/bdrc.21117 26663884

[B25] Koren O. Goodrich J. K. Cullender T. C. Spor A. Laitinen K. Bäckhed H. K.. (2012). Host Remodeling of the Gut Microbiome and Metabolic Changes During Pregnancy. Cell 150 (3), 470–480. doi: 10.1016/j.cell.2012.07.008 22863002 PMC3505857

[B26] Liu H.. (2018). The Family Coriobacteriaceae Is a Potential Contributor to the Beneficial Effects of Roux-En-Y Gastric Bypass on Type 2 Diabetes. Surg. Obes. Related Dis: Off. J. Am. Soc. Bariatric Surg. 14 (5), 584–593. doi: 10.1016/j.soard.2018.01.012 29459013

[B27] Lui N. A. Jeyaram G. Henry A. (2019). Postpartum Interventions to Reduce Long-Term Cardiovascular Disease Risk in Women After Hypertensive Disorders of Pregnancy: A Systematic Review. Front. Cardiovasc. Med. 6, 160–160. doi: 10.3389/fcvm.2019.00160 31803757 PMC6873287

[B28] Lv L.-J. Sheng-Hui L. Shao-Chuan L. Zhi-Cheng Z. Hong-Li D. Tian C.. (2019). Early-Onset Preeclampsia Is Associated With Gut Microbial Alterations in Antepartum and Postpartum Women. Front. Cell. Infect. Microbiol. 9, 224–224. doi: 10.3389/fcimb.2019.00224 31297341 PMC6608563

[B29] Maher G. M. O’Keeffe G. W. Kearney P. M. Kenny L. C. Dinan T. G. Mattsson M.. (2018). Association of Hypertensive Disorders of Pregnancy With Risk of Neurodevelopmental Disorders in Offspring: A Systematic Review and Meta-Analysis. JAMA Psychiatry 75, (8), 809–819. doi: 10.1001/jamapsychiatry.2018.0854 29874359 PMC6143097

[B30] Makino H. Kushiro A. Ishikawa E. Kubota H. Gawad A. Sakai T.. (2013). Mother-to-Infant Transmission of Intestinal Bifidobacterial Strains Has an Impact on the Early Development of Vaginally Delivered Infant’s Microbiota. PLoS ONE 8, (11), e78331. doi: 10.1371/journal.pone.0078331 24244304 PMC3828338

[B31] Marcobal A. Sonnenburg J. L. (2012). Human Milk Oligosaccharide Consumption by Intestinal Microbiota. Clin. Microbiol. Infection: Off. Publ. Eur. Soc. Clin. Microbiol. Infect. Dis. 18 Suppl 4 (0 4), 12–15. doi: 10.1111/j.1469-0691.2012.03863.x PMC3671919 22647041

[B32] McLennan S. L. Henry A. Roberts L. M. Siritharan S. S. Ojurovic M. Yao A.. (2021). Maternal Adiposity and Energy Balance After Normotensive and Preeclamptic Pregnancies. J. Clin. Endocrinol. Metab. 106 (8), e2941–e2952. doi: 10.1210/clinem/dgab223 PMC8277202 33824990

[B33] McMurdie P. J. Holmes S. (2013). Phyloseq: An R Package for Reproducible Interactive Analysis and Graphics of Microbiome Census Data. PloS One 8 (4), e61217. doi: 10.1371/journal.pone.0061217 23630581 PMC3632530

[B34] Nuriel-Ohayon M. Neuman H. Ziv O. Belogolovski A. Barsheshet Y. Bloch N.. (2019). Progesterone Increases Bifidobacterium Relative Abundance During Late Pregnancy. Cell Rep. 27 (3), 730–736.e3. doi: 10.1016/j.celrep.2019.03.075 30995472

[B35] Parada Venegas D.. (2019). Short Chain Fatty Acids (SCFAs)-Mediated Gut Epithelial and Immune Regulation and Its Relevance for Inflammatory Bowel Diseases. Front. Immunol. 10, 277. doi: 10.3389/fimmu.2019.00277 30915065 PMC6421268

[B36] Penders J. Thijs C. Vink C. Stelma F. F. Snijders B. Kummeling I.. (2006). Factors Influencing the Composition of the Intestinal Microbiota in Early Infancy. Pediatrics 118 (2), 511–521. doi: 10.1542/peds.2005-2824 16882802

[B37] Pimenta F. Gertz R. E. Jr. Park S. H. Kim E. Moura I. Milucky J.. (2019). Streptococcus Infantis, Streptococcus Mitis, and Streptococcus Oralis Strains With Highly Similar Cps5 Loci and Antigenic Relatedness to Serotype 5 Pneumococci. Front. Microbiol. 9, 3199–3199. doi: 10.3389/fmicb.2018.03199 30671034 PMC6332807

[B38] R Core Team. (2020). R: A language and environment for statistical computing. R Foundation for Statistical Computing, (Vienna, Austria). Available at: https://www.R-project.org/.

[B39] RStudio Team. (2020). RStudio. Integrated Development for R (Boston, MA: RStudio, PBC). Available at: http://www.rstudio.com/.

[B40] Segata N. Izard J. Waldron L. Gevers D. Miropolsky L. Garrett W. S.. (2011). Metagenomic Biomarker Discovery and Explanation. Genome Biol. 12, R60. doi: 10.1186/gb-2011-12-6-r60 21702898 PMC3218848

[B41] Siritharan S. S. Henry A. Gow M. L. Roberts L. M. Yao A. Ojurovic M.. (2021). Maternal Macro- and Micronutrient Intake Six Months After Hypertensive Versus Normotensive Pregnancy: Is Poor Diet Quality Contributing to Future Cardiometabolic Disease Risk? Pregnancy Hypertension 23, 196–204. doi: 10.1016/j.preghy.2020.11.002 33515976

[B42] Susic D. Davis G. O' Sullivan A. J. McGovern E. Harris K. Roberts L. M.. (2020). Microbiome Understanding in Maternity Study (MUMS), an Australian Prospective Longitudinal Cohort Study of Maternal and Infant Microbiota: Study Protocol. BMJ Open 10 (9), e040189. doi: 10.1136/bmjopen-2020-040189 PMC7493111 32933964

[B43] Tenenbaum-Gavish K. Sharabi-Nov A. Binyamin D. Møller H. J. Danon D. Rothman L.. (2020). First Trimester Biomarkers for Prediction of Gestational Diabetes Mellitus. Placenta 101, 80–89. doi: 10.1016/j.placenta.2020.08.020 32937245

[B44] Theilen L. H. Fraser A. Hollingshaus M. S. Schliep K. C. Varner M. W. Smith K. R.. (2016). All-Cause and Cause-Specific Mortality After Hypertensive Disease of Pregnancy. Obstet. Gynecol. 128 (2), 238–244. doi: 10.1097/AOG.0000000000001534 27400006 PMC4961555

[B45] Underwood M. A. Davis J. C.C. Kalanetra K. M. Gehlot S. Patole S. Tancredi D. J.. (2017). Digestion of Human Milk Oligosaccharides by Bifidobacterium Breve in the Premature Infant. J. Pediatr. Gastroenterol. Nutr. 65 (4), 449–455. doi: 10.1097/MPG.0000000000001590 28945208 PMC6349036

[B46] van der Giessen J. Binyamin D. Belogolovski A. Frishman S. Tenenbaum-Gavish K. Hadar E.. (2020). Modulation of Cytokine Patterns and Microbiome During Pregnancy in IBD. Gut 69 (3), 473. doi: 10.1136/gutjnl-2019-318263 31167813 PMC7034354

[B47] Vatanen T. Kostic A. D. d'Hennezel E. Siljander H. Franzosa E. A. Yassour M.. (2016). Variation in Microbiome LPS Immunogenicity Contributes to Autoimmunity in Humans. Cell 165 (4), 842–853. doi: 10.1016/j.cell.2016.04.007 27133167 PMC4950857

[B48] Wang L. Christophersen C. T. Sorich M. J. Gerber J. P. Angley M. T. Conlon M. A.. (2013). Increased Abundance of Sutterella Spp. And Ruminococcus Torques in Feces of Children With Autism Spectrum Disorder. Mol. Autism 4 (1), 42. doi: 10.1186/2040-2392-4-42 24188502 PMC3828002

[B49] Wang J. Zheng J. Shi W. Du N. Xu X. Zhang Y.. (2018). Dysbiosis of Maternal and Neonatal Microbiota Associated With Gestational Diabetes Mellitus. Gut 67 (9), 1614–1625. doi: 10.1136/gutjnl-2018-315988 29760169 PMC6109274

[B50] Wickham H. (2016). “Elegant Graphics for Data Analysis,” in Ggplot2, 2 ed, vol. XVI. (Houston, TX: Springer International Publishing), 260.

[B51] Wickham H. François R. Henry L. Müller K. (2019) Dplyr: A Grammar of Data Manipulation, R.F. Available at: https://CRAN.R-project.org/package=dplyr.

[B52] Wu P. Haththotuwa R. Kwok C. S. Babu A. Kotronias R. A. Rushton C.. (2017). Preeclampsia and Future Cardiovascular Health: A Systematic Review and Meta-Analysis. Circ. Cardiovasc. Qual. Outcomes 10 (2), e003497. doi: 10.1161/CIRCOUTCOMES.116.003497 28228456

