# Corrigendum: The P4 Study: Postpartum Maternal and Infant Faecal Microbiome 6 Months After Hypertensive Versus Normotensive Pregnancy

**DOI:** 10.3389/fcimb.2022.895332

**Published:** 2022-03-31

**Authors:** Daniella Frances Susic, Leanne Wang, Lynne Margaret Roberts, Michelle Bai, Andrew Gia, Emily McGovern, Xiao-Tao Jiang, Gregory K. Davis, Emad El-Omar, Amanda Henry

**Affiliations:** ^1^School of Women’s and Children’s Health, Faculty of Medicine, University of New South Wales, Sydney, NSW, Australia; ^2^Microbiome Research Centre, University of New South Wales, Sydney, NSW, Australia; ^3^Department of Women’s and Children’s Health, St. George Hospital, Sydney, NSW, Australia; ^4^Faculty of Medicine, University of New South Wales, Sydney, NSW, Australia; ^5^St. George and Sutherland Clinical School, University of New South Wales, Sydney, NSW, Australia; ^6^George Institute for Global Health, University of New South Wales, Sydney, NSW, Australia

**Keywords:** pregnancy, infancy, microbiome, preeclampsia, hypertensive pregnancy, postpartum

In the original article within *Discussion, paragraph 6, sentence 4*, the incorrect citation of “Getahun et al., 2017” was used instead of the correct citation “Maher et al., 2018” and the word “preeclampsia” was used instead of “hypertensive disorder”. The corrected sentence reads: “Wang et al. found increased *Sutterella* species in children who went on to develop autism spectrum disorder (Wang et al., 2013) and a meta-analysis performed by Maher et al. showed a 35% increase in the odds of having a child with autism in hypertensive disorder exposed pregnancies (Maher et al., 2018)”.

The full reference for “Getahun et al., 2017” has been removed from the reference list and replaced with “Maher, G. M., O’Keeffe, G. W., Kearney, P. M., Kenny, L. C., Dinan, T. G., Mattsson, M., et al. (2018). Association of Hypertensive Disorders of Pregnancy With Risk of Neurodevelopmental Disorders in Offspring: A Systematic Review and Meta-analysis. *JAMA psychiatry*, *75*(8), 809–819. https://doi.org/10.1001/jamapsychiatry.2018.0854”.

The authors apologize for this error and state that this does not change the scientific conclusions of the article in any way. The original article has been updated.

In the original article, there was an error in the spelling of several bacterial names which may impair the clear interpretation of findings.

A correction to the spelling of *Barnesiella* and *Bifidobacterium* sp. has been made to *Abstract*, sub-section *Results, sentence 8*. The updated sentence reads: ***“*** It was also found that at a genus and species level, the gut microbiota of HP women was enriched with *Bifidobacterium* and *Bifidobacterium* sp. and depleted in *Barnesiella* and *Barnesiella intestinihominis* when compared to NP women (*P* < 0.05)”.

A correction to the spelling of *Bifidobacterium* sp. has been made to *Results*, sub-section *Changes in Gut Microbiota Composition Between Women After HP and NP*, *sentence 3*. The updated sentence reads: “The gut microbiota of HP women was enriched in phylum Actinobacteria, order Bifidobacteriales, family Bifidobacteriaceae, genus *Bifidobacterium* and species *Bifidobacterium* sp. compared to NP women (LDA > 2, *P* < 0.05)”.

A correction to the spelling of *Streptococcus infantis* has been made to *Discussion, paragraph 6, sentence 1.* The updated sentence reads: “In this study, one enriched species of bacteria from the Firmicutes phylum, *Streptococcus infantis* was found in the infants born from HP mothers”.

The authors apologize for this error and state that this does not change the scientific conclusions of the article in any way. The original article has been updated.

## Publisher’s Note

All claims expressed in this article are solely those of the authors and do not necessarily represent those of their affiliated organizations, or those of the publisher, the editors and the reviewers. Any product that may be evaluated in this article, or claim that may be made by its manufacturer, is not guaranteed or endorsed by the publisher.
