# Supplementary material for: The P4 Study: Postpartum Maternal and Infant Faecal Microbiome 6 Months After Hypertensive Versus Normotensive Pregnancy
Source: Front Cell Infect Microbiol. 2022 Jan 31;12:646165. doi: 10.3389/fcimb.2022.646165 (PMC8860159; doi:10.3389/fcimb.2022.646165)
Supplement: Supplementary file 1 [file DataSheet_1.pdf]

Supplementary Materials

Supplementary Table 1: Alpha diversity comparison between mothers and babies

Alpha Diversity Index	Baby (*n* = 18)	Mum (*n* = 18)	*P* value
Faith's PD	13.255	34.673	**8.82E-10**
Shannon's Entropy	4.364	6.335	**1.76E-09**
Observed OTUs	107.611	382.778	**4.29E-07**
Pielou's Evenness	0.651	0.741	**2.87E-05**

Abbreviations: PD, phylogenetic diversity; OTU, operational taxonomic unit.
Results are presented as mean ± standard deviation.
Significant *P* values calculated from Wilcoxon rank sum test and Benjamini Hochberg correction are in bold.

Supplementary Table 2: PERMANOVA of beta diversity in mothers and babies based on Bray Curtis dissimilarities

Groups	Df	SS	F Model	R^2	*P* value
Mothers vs Babies	1	3.987	22.497	0.398	**0.001**

Abbreviations: Df, degrees of freedom; SS, sum of squares; F Model, F value by permutation; R^2, effect size.
Significant *P* values from PERMANOVA with Benjamini Hochberg correction based on 999 permutations are in bold.

Supplementary Table 3: Alpha diversity comparison between HP and NP mothers

Alpha Diversity Index	HP Mum (*n* = 8)	NP Mum (*n* = 10)	*P* value
Faith's PD	36.203	33.449	0.897
Shannon's Entropy	6.288	6.372	0.897
Observed OTUs	377.625	386.900	0.897
Pielou's Evenness	0.738	0.744	0.897

Abbreviations: PD, phylogenetic diversity; OTU, operational taxonomic unit; HP, hypertensive pregnancy; NP, normotensive pregnancy.
Results are presented as mean ± standard deviation.
P values are calculated from Wilcoxon rank sum test with Benjamini Hochberg correction.

Supplementary Table 4: PERMANOVA of beta diversity in HP and NP mothers based on Bray Curtis dissimilarities

Groups	Df	SS	F Model	R^2	*P* value
Mum HP vs Mum NP	1	0.110	0.859	0.051	0.617

Abbreviations: Df, degrees of freedom; SS, sum of squares; F Model, F value by permutation; R^2, effect size; HP, hypertensive pregnancy; NP, normotensive pregnancy; PERMANOVA, permutational multivariate analysis of variance.
P values are calculated from PERMANOVA with Benjamini Hochberg correction based on 999 permutations.

Supplementary Table 5: Differential taxa between HP and NP mothers using LEfSe from phylum to species level

Taxa	Group with higher relative abundance	LDA effect size	LDA effect size Revised	Unadjusted *P* value	FDR adjusted *P* value
g_Bifidobacterium	HP Mum	2.399	-2.399	**0.002**	0.342
c_Actinobacteria	HP Mum	2.399	-2.399	**0.003**	0.342
p_Actinobacteria	HP Mum	2.423	-2.423	**0.016**	0.693
f_Bifidobacteriaceae	HP Mum	2.399	-2.399	**0.002**	0.342
s_Bifidobacterium_unclassified	HP Mum	2.111	-2.111	**0.026**	0.693
g_Barnesiella	NP Mum	2.498	2.498	**0.022**	0.693
o_Bifidobacteriales	HP Mum	2.399	-2.399	**0.002**	0.342
s_Barnesiella_intestinihominis	NP Mum	2.498	2.498	**0.022**	0.693

Abbreviations: LDA, linear discriminant analysis; LEfSe, Linear discriminant analysis Effect Size; HP, hypertensive pregnancy, NP; normotensive pregnancy.
P values from LEfSe are presented as both unadjusted and adjusted with Benjamini Hochberg correction. Significant *P* values are in bold.

Supplementary Table 6: Alpha diversity comparison between mothers based on smoking status

Alpha Diversity Index	Mum No Smoking (*n* = 13)	Mum Yes Smoking (*n* = 5)	*P* value
Shannon's entropy	6.308 ± 0.533	6.404 ± 0.5560	1
Faith's PD	34.484 ± 9.314	35.163 ± 4.546	1
Pielou's evenness	0.741 ± 0.030	0.742 ± 0.048	1
Observed OTUs	376.615 ± 105.002	398.8 ± 62.432	1

Abbreviations: PD, phylogenetic diversity; OTU, operational taxonomic unit.
Results are presented as mean ± standard deviation.
P values are calculated from Wilcoxon rank sum test with Benjamini Hochberg correction.

Supplementary Table 7: Alpha diversity comparison between mothers based on alcohol intake

Alpha Diversity Index	Mum No Alcohol (*n* = 10)	Mum Yes Alcohol (*n* = 8)	*P* value
Observed OTUs	392.800 ± 92.471	370.250 ± 100.325	1
Shannon's entropy	6.344 ± 0.507	6.323 ± 0.583	1
Faith's PD	34.455 ± 8.866	34.945 ± 7.717	1
Pielou's evenness	0.739 ± 0.030	0.744 ± 0.041	1

Abbreviations: PD, phylogenetic diversity; OTU, operational taxonomic unit.
Results are presented as mean ± standard deviation.
P values are calculated from Wilcoxon rank sum test with Benjamini Hochberg correction.

Supplementary Table 8: Alpha diversity comparison between mothers based on multivitamin intake

Alpha Diversity Indices	Mum No Multivitamin (*n* = 12)	Mum Yes Multivitamin (*n* = 6)	*P* value
Observed OTUs	343.333 ± 91.735	461.667 ± 14.787	**0.040***
Shannon's entropy	6.128 ± 0.526	6.748 ± 0.149	0.071

Pielou's evenness	0.731 ± 0.036	0.763 ± 0.019	0.071
Faith's PD	32.432 ± 7.888	39.156 ± 7.213	0.125

Abbreviations: PD, phylogenetic diversity; OTU, operational taxonomic unit.
Results are presented as mean ± standard deviation.
Significant *P* values calculated from Wilcoxon rank sum test with Benjamini Hochberg correction are in bold.

Supplementary Table 9: PERMANOVA of beta diversity in mothers grouped according to clinical variables and based on Bray Curtis dissimilarities

Groups	Df	SS	F Model	R^2	*P* value
Mum No Multivitamin vs Mum Yes Multivitamin	1	0.236	1.967	0.109	**0.035***
Mum No Smoking vs Mum Yes Smoking	1	0.193	1.580	0.090	0.083
Mum No Alcohol vs Mum Yes Alcohol	1	0.130	1.030	0.060	0.418

Abbreviations: Df, degrees of freedom; SS, sum of squares; F Model, F value by permutation; R^2, effect size; PERMANOVA, permutational multivariate analysis of variance. Significant *P* values from PERMANOVA with Benjamini Hochberg correction based on 999 permutations are in bold.

Supplementary Table 10: Differential genera in mothers based on multivitamin intake in gestation and postpartum

Taxa	Group with Higher Relative Abundance	LDA Effect Size	LDA Effect Size Revised	Unadjusted *P* value	FDR adjusted *P* value
g_AF12	Yes Multivitamin	2.407	2.407	**0.040**	0.386
g_Campylobacter	Yes Multivitamin	2.697	2.697	**0.019**	0.386
g_Bacteroides	No Multivitamin	4.923	-4.923	**0.031**	0.386
g_unclassified_o_Clostridiales	Yes Multivitamin	4.243	4.243	**0.019**	0.386
g_Butyricicoccus	No Multivitamin	3.104	-3.104	**0.007**	0.386
g_Synergistes	Yes Multivitamin	2.968	2.968	**0.040**	0.386
g_Lachnobacterium	Yes Multivitamin	3.227	3.227	**0.034**	0.386
g_unclassified_f_Mogibacteriaceae	Yes Multivitamin	2.912	2.912	**0.025**	0.386
g_unclassified_f_Ruminococcaceae	Yes Multivitamin	4.293	4.293	**0.011**	0.386
g_Neisseria	Yes Multivitamin	2.786	2.786	**0.040**	0.386
g_Catenibacterium	Yes Multivitamin	2.905	2.905	**0.040**	0.386
g_unclassified_o_Streptophyta	Yes Multivitamin	2.295	2.295	**0.013**	0.386

g_Oscillospira	Yes Multivitamin	4.173	4.173	**0.039**	0.386
g_unclassified_o_RF39	Yes Multivitamin	2.961	2.961	**0.019**	0.386
g_unclassified_f_Cerasicoccaceae	Yes Multivitamin	2.805	2.805	**0.006**	0.386
g_unclassified_f_Coriobacteriaceae	Yes Multivitamin	2.610	2.610	**0.037**	0.386

Abbreviations: LDA, linear discriminant analysis; LEfSe, Linear discriminant analysis Effect Size.
P values from LEfSe are presented as both unadjusted and adjusted with Benjamini Hochberg correction. Significant *P* values are in bold.

Supplementary Table 11: Differential species in mothers based on multivitamin intake in gestation and postpartum

Taxa	Group with Higher Relative Abundance	LDA Effect Size	LDA Effect Size Revised	Unadjusted *P* value	FDR adjusted *P* value
s_Synergistaceae_unclassified	Yes Multivitamin	3.018	3.018	**0.040**	0.510
s_unclassified_f_Mogibacteriaceae	Yes Multivitamin	2.993	2.993	**0.025**	0.510
s_AF12_unclassified	Yes Multivitamin	3.669	3.669	**0.040**	0.510
s_Oscillospira_unclassified	Yes Multivitamin	4.177	4.177	**0.039**	0.510
s_Bacteroides_ovatus	No Multivitamin	4.204	-4.204	**0.031**	0.510
s_Catenibacterium_unclassified	Yes Multivitamin	2.920	2.920	**0.040**	0.510
s_Ruminococcus_torques	No Multivitamin	3.160	-3.160	**0.025**	0.510
s_unclassified_f_Cerasicoccaceae	Yes Multivitamin	2.946	2.946	**0.006**	0.510
s_Lachnobacterium_unclassified	Yes Multivitamin	3.247	3.247	**0.034**	0.510
s_unclassified_f_Ruminococcaceae	Yes Multivitamin	4.293	4.293	**0.011**	0.510
s_unclassified_o_Clostridiales	Yes Multivitamin	4.243	4.243	**0.019**	0.510
s_unclassified_o_RF39	Yes Multivitamin	3.059	3.059	**0.019**	0.510
s_unclassified_f_Coriobacteriaceae	Yes Multivitamin	2.946	2.946	**0.037**	0.510
s_Campylobacter_unclassified	Yes Multivitamin	3.505	3.505	**0.014**	0.510
s_unclassified_o_Streptophyta	Yes Multivitamin	3.451	3.451	**0.013**	0.510

s_Butyricicoccus_pullicaecorum	No Multivitamin	3.154	-3.154	**0.007**	0.510
s_Bacteroides_plebeius	No Multivitamin	3.887	-3.887	**0.006**	0.510
s_Bacteroides_uniformis	No Multivitamin	4.342	-4.342	**0.039**	0.510

Abbreviations: LDA, linear discriminant analysis; LEfSe, Linear discriminant analysis Effect Size.
P values from LEfSe are presented as both unadjusted and adjusted with Benjamini Hochberg correction. Significant *P* values are in bold.

Supplementary Table 12: Alpha diversity comparison between HP and NP babies

Alpha Diversity Index	HP Baby (*n* = 8)	NP Baby (*n* = 10)	*P* value
Shannon's entropy	3.920	4.719	**0.031**
Pielou's evenness	0.608	0.685	**0.031**
Observed OTUs	90.375	121.400	0.075
Faith's PD	12.800	13.618	0.897

Abbreviations: PD, phylogenetic diversity; HP, hypertensive pregnancy; NP, normotensive pregnancy; OTU, operational taxonomic unit.
Results are presented as mean ± standard deviation.
Significant *P* values calculated from Wilcoxon rank sum test and Benjamini Hochberg correction are in bold.

Supplementary Table 13: PERMANOVA of beta diversity in HP and NP babies on Bray Curtis dissimilarities

Groups	Df	SS	F Model	R^2	*P* value
HP Baby vs NP Baby	1	0.266	1.181	0. 069	0.291

Abbreviations: Df, degrees of freedom; SS, sum of squares; F Model, F value by permutation; R^2, effect size; HP, hypertensive pregnancy; NP, normotensive pregnancy.
P values are from PERMANOVA with Benjamini Hochberg correction based on 999 permutations.

Supplementary Table 14: Differential taxa between HP and NP babies using LEfSe

Taxa	Group with Higher Relative Abundance	LDA effect size	LDA effect size Revised	Unadjusted *P* value	FDR adjusted *P* value
s_Clostridium_aldenense	NP Baby	2.919	2.919	**0.025**	0.590
f_Coriobacteriaceae	NP Baby	2.336	2.336	**0.046**	0.590
o_Coriobacteriales	NP Baby	2.336	2.336	**0.046**	0.590
s_Bacteroides_unclassified	NP Baby	4.172	4.172	**0.029**	0.590
c_Betaproteobacteria	NP Baby	2.980	2.980	**0.037**	0.590
s_Sutterella_unclassified	NP Baby	3.020	3.020	**0.034**	0.590
s_Streptococcus_infantis	HP Baby	2.030	-2.030	**0.037**	0.590
c_Coriobacteriia	NP Baby	2.336	2.336	**0.046**	0.590
p_Bacteroidetes	NP Baby	4.239	4.239	**0.033**	0.590
c_Bacteroidia	NP Baby	4.227	4.227	**0.033**	0.590

o_Bacteroidales	NP Baby	4.227	4.227	**0.033**	0.590
f_Alcaligenaceae	NP Baby	3.305	3.305	**0.034**	0.590
g_Sutterella	NP Baby	3.285	3.285	**0.034**	0.590
o_Burkholderiales	NP Baby	3.071	3.071	**0.017**	0.590

Abbreviations: LDA, linear discriminant analysis; LEfSe, Linear discriminant analysis Effect Size; NP, normotensive pregnancy; HP, hypertensive pregnancy.
P values from LEfSe are presented as both unadjusted and adjusted with Benjamini Hochberg correction. Significant *P* values are in bold.

Supplementary Table 15: Alpha diversity comparison between babies based on maternal smoking status

Alpha Diversity Index	Baby (Mum No Smoking) (*n* = 13)	Baby (Mum Yes Smoking) (*n* = 5)	*P* value
Shannon's entropy	4.299 ± 0.664	4.532 ± 0.713	0.730
Faith's PD	12.883 ± 4.945	14.221 ± 5.097	0.730
Pielou's evenness	0.644 ± 0.059	0.670 ± 0.085	0.730
Observed OTUs	106.923 ± 38.776	109.4 ± 21.801	0.730

Abbreviations: PD, phylogenetic diversity; OTU, operational taxonomic unit.
Results are presented as mean ± standard deviation.
P values are calculated from Wilcoxon rank sum test with Benjamini Hochberg correction.

Supplementary Table 16: Alpha diversity comparison between babies based on maternal alcohol intake

Alpha Diversity Index	Baby (Mum No Alcohol) (*n* = 10)	Baby (Mum Yes Alcohol) (*n* = 8)	*P* value
Observed OTUs	125.300 ± 31.411	85.500 ± 24.042	0.058
Shannon's entropy	4.659 ± 0.665	3.995 ± 0.476	0.109
Faith's PD	14.788 ± 4.351	11.338 ± 5.080	0.194
Pielou's evenness	0.671 ± 0.074	0.626 ± 0.046	0.408

Abbreviations: PD, phylogenetic diversity; OTU, operational taxonomic unit.
Results are presented as mean ± standard deviation.
P values are calculated from Wilcoxon rank sum test with Benjamini Hochberg correction.

Supplementary Table 17: Alpha diversity comparison between babies based on birth method

Alpha Diversity Index	Caesarean Section Delivery Baby (*n* = 4)	Vaginal Delivery Baby (*n* =14)	*P* value
Pielou's evenness	0.611 ± 0.041	0.662 ± 0.068	0.507
Faith's PD	10.763 ± 4.103	13.967 ± 4.972	0.554
Shannon entropy	4.057 ± 0.591	4.452 ± 0.679	0.674
Observed OTUs	103.000 ± 34.029	108.929 ± 35.500	0.958

Abbreviations: PD, Phylogenetic Diversity; OTU, operational taxonomic unit.
Results are presented as mean ± standard deviation.
P values are calculated from Wilcoxon rank sum test with Benjamini Hochberg correction.

Supplementary Table 18: Alpha diversity comparison between babies based on feeding method

Alpha Diversity Index	Breast Feeding (*n* = 8)	Bottle Feeding (*n* = 9)	Mix Feeding (*n* = 1)	*P* value Breast-feeding vs Bottle-Feeding	*P* value Breast-feeding vs Mix-Feeding	*P* value Bottle-Feeding vs Mix-Feeding
Observed OTUs	95.625 ± 26.076	130.000 ± 40.242	115.778	0.411	0.225	0.267
Pielou's evenness	0.636 ± 0.064	0.627 ± 0.071	0.666	0.329	0.570	0.297
Shannon's entropy	4.172 ± 0.650	4.406 ± 0.708	4.531	0.568	0.561	0.461
Faith's PD	14.049 ± 5.505	14.390 ± 4.733	12.422	0.456	0.725	0.498

Abbreviations: PD, Phylogenetic Diversity; OTU, operational taxonomic unit.
Results are presented as mean ± standard deviation.
P values are calculated from Kruskal-Wallis test with Dunn's posthoc test and Benjamini Hochberg correction.

Supplementary Table 19: Alpha diversity comparison in babies based on maternal multivitamin intake during pregnancy

Alpha Diversity Index	Baby (Mum No Multivitamin) (*n* = 12)	Baby (Mum Yes Multivitamin) (*n* = 6)	*P* value
Observed OTUs	107.083 ± 39.583	108.667 ± 23.330	0.964
Shannon's entropy	4.317 ± 0.680	4.458 ± 0.688	0.964
Pielou's evenness	0.646 ± 0.060	0.660 ± 0.080	0.964
Faith's PD	12.140 ± 4.693	15.470 ± 4.847	0.852

Abbreviations: PD, Phylogenetic Diversity; OTU, operational taxonomic unit.
Results are presented as mean ± standard deviation.
P values are calculated from Wilcoxon rank sum test with Benjamini Hochberg correction.

Supplementary Table 20: PERMANOVA of beta diversity in babies based on Bray Curtis dissimilarities

Pair	Df	SS	F Model	R^2	*P* value
Vaginal vs Caesarean-Section Delivery	1	0.394	1.811	0.102	0.079
Mix Feeding vs Breast Feeding	1	0.404	1.828	0.109	0.213
Mix Feeding vs Bottle Feeding	1	0.218	0.882	0.099	1.000
Breast Feeding vs Bottle Feeding	1	0.118	0.617	0.081	1.000
Baby (Mum No Smoking) vs Baby (Mum Yes Smoking)	1	0.214	0.934	0.055	0.455
Baby (Mum No Alcohol) vs Baby (Mum Yes Alcohol)	1	0.131	0.559	0.034	0.820
Baby (Mum No Multivitamin) vs Baby (Mum Yes Multivitamin)	1	0.074	0.311	0.019	0.975

Abbreviations: Df, degrees of freedom; SS, sum of squares; F Model, F value by permutation; R^2, effect size; PERMANOVA, permutational multivariate analysis of variance. *P* values are from PERMANOVA with Benjamini Hochberg correction based on 999 permutations.

Supplementary Table 21: Differential genera between babies grouped by maternal multivitamin intake in pregnancy using LEfSe

Taxa	Group with Higher Relative Abundance	LDA Effect Size	LDA Effect Size Revised	Unadjusted *P* value	FDR adjusted *P* value
g_Megasphaera	Yes Multivitamin	3.386	3.386	**0.006**	0.701
g_Eubacterium	No Multivitamin	3.294	-3.294	**0.045**	0.701

Abbreviations: LDA, linear discriminant analysis; LEfSe, Linear discriminant analysis Effect Size.
P values from LEfSe are presented as both unadjusted and adjusted with Benjamini Hochberg correction. Significant *P* values are in bold.

Supplementary Table 22: Differential species between babies based grouped by maternal multivitamin intake in pregnancy using LEfSe

Taxa	Group with Higher Relative Abundance	LDA Effect Size	LDA Effect Size Revised	Unadjusted *P* value	FDR adjusted *P* value
s_Eubacterium_dolichum	No Multivitamin	3.273	-3.273	**0.045**	0.669
s_Megasphaera_unclassified	Yes Multivitamin	3.598	3.5977	**0.006**	0.669
s_Actinobacillus_parahaemolyticus	Yes Multivitamin	3.569	3.569	**0.040**	0.669
s_Clostridium_unclassified	No Multivitamin	3.877	-3.877	**0.045**	0.669

Abbreviations: LDA, linear discriminant analysis; LEfSe, Linear discriminant analysis Effect Size.
P values from LEfSe are presented as both unadjusted and adjusted with Benjamini Hochberg correction. Significant *P* values are in bold.

Supplementary Figure 1

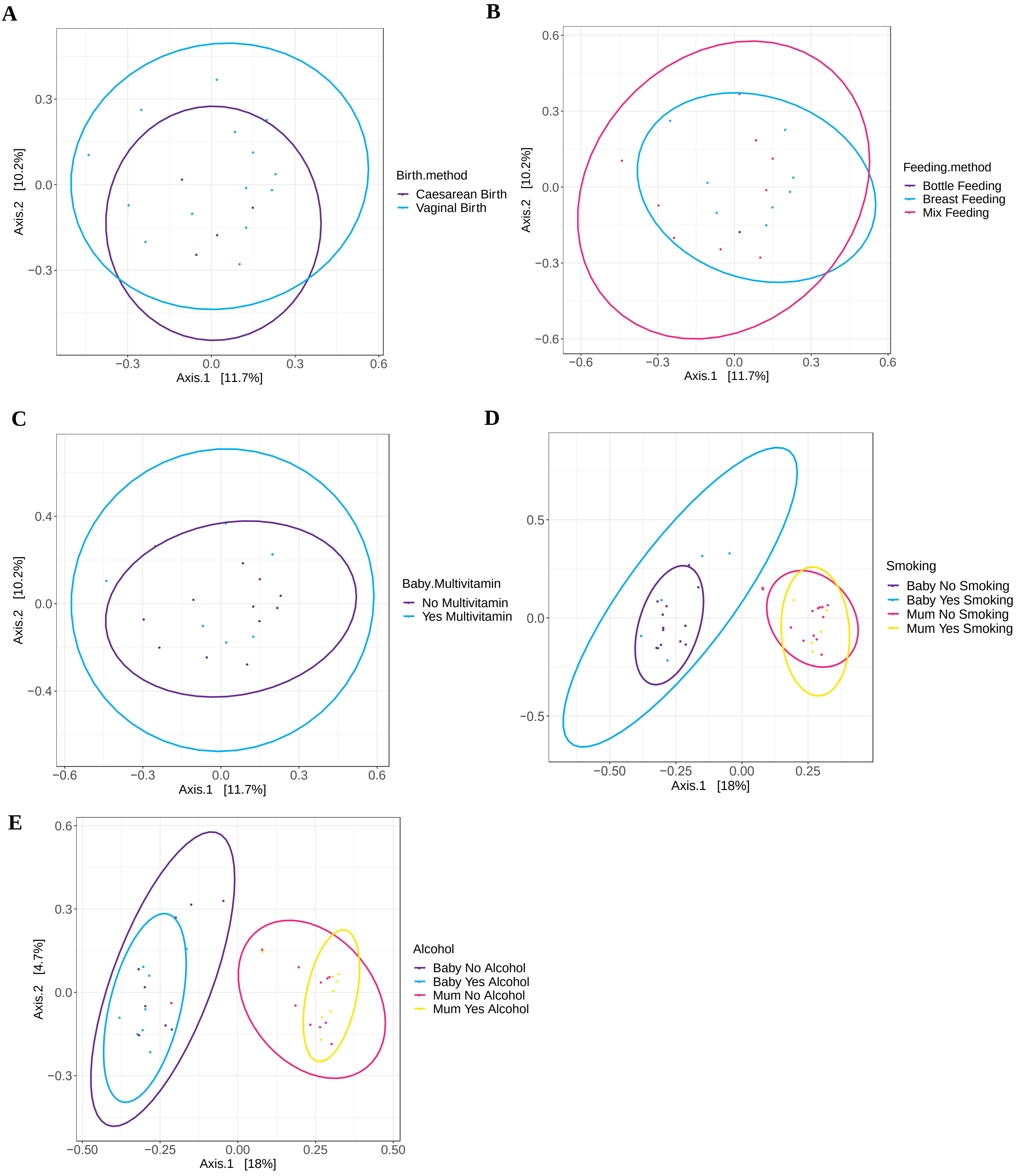

Supplementary Figure 1. Principle coordinate analysis (PCoA) plot of microbial communities in mothers and babies. **(A)** PCoA of Bray Curtis distances between babies based on birth method. **(B)** PCoA of Bray Curtis distances between babies based on feeding method. **(C)** PCoA of Bray Curtis distances between babies based on maternal multivitamin intake in pregnancy. **(D)** PCoA of Bray Curtis distances between mothers based on pre-pregnancy and postpartum smoking status, and in babies based on maternal smoking status. **(E)** PCoA of Bray Curtis distances between mothers based on pre-pregnancy and post-partum alcohol intake, and in babies based on maternal alcohol intake. Data points represent individual samples and are colour coded accorded their groups. Ellipses indicate the 95% confidence intervals of multivariate normal distribution. The distance between points is representative of similarity or dissimilarity in microbiota composition between samples. The percentage value of each axis represents the amounts of variation it explains. Overall the lack of clustering of points based on each factor assessed indicates that microbiota composition in babies is not significantly grouped according to birth method, feeding method or multivitamin intake. Additionally, microbiota composition in both mothers and babies are not significantly segregated according to smoking status or alcohol intake.
